# Dietary fat restriction affects brain reward regions in a randomized crossover trial

**DOI:** 10.1172/jci.insight.169759

**Published:** 2023-06-22

**Authors:** Valerie L. Darcey, Juen Guo, Amber B. Courville, Isabelle Gallagher, Jason A. Avery, W. Kyle Simmons, John E. Ingeholm, Peter Herscovitch, Alex Martin, Kevin D. Hall

**Affiliations:** 1 Integrative Physiology Section, National Institute of Diabetes & Digestive & Kidney Diseases, Bethesda, Maryland, USA.; 2 Human Energy and Body Weight Regulation Core, National Institute of Diabetes & Digestive & Kidney Diseases, Bethesda, Maryland, USA.; 3 Laboratory of Brain and Cognition, National Institute of Mental Health, Rockland, Maryland, USA.; 4 Biomedical Imaging Center, Oklahoma State University, Stillwater, Oklahoma, USA.; 5 Clinical Center Positron Emission Tomography Department, NIH, Bethesda, Maryland, USA.

**Keywords:** Metabolism, Neuroscience, Obesity

## Abstract

**BACKGROUND:**

Weight-loss diets often target dietary fat or carbohydrates, macronutrients that are sensed via distinct gut-brain pathways and differentially affect peripheral hormones and metabolism. However, the effects of such diet changes on the human brain are unclear.

**METHODS:**

We investigated whether selective isocaloric reductions in dietary fat or carbohydrates altered dopamine D2/3 receptor binding potential (D2BP) and neural activity in brain-reward regions in response to visual food cues in 17 inpatient adults with obesity as compared with a eucaloric baseline diet using a randomized crossover design.

**RESULTS:**

On the fifth day of dietary fat restriction, but not carbohydrate restriction, both D2BP and neural activity to food cues were decreased in brain-reward regions. After the reduced-fat diet, ad libitum intake shifted toward foods high in both fat and carbohydrates.

**CONCLUSION:**

These results suggest that dietary fat restriction increases tonic dopamine in brain-reward regions and affects food choice in ways that may hamper diet adherence.

**TRIAL REGISTRATION:**

ClinicalTrials.gov NCT00846040 FUNDING. NIDDK 1ZIADK013037.

## Graphical abstract

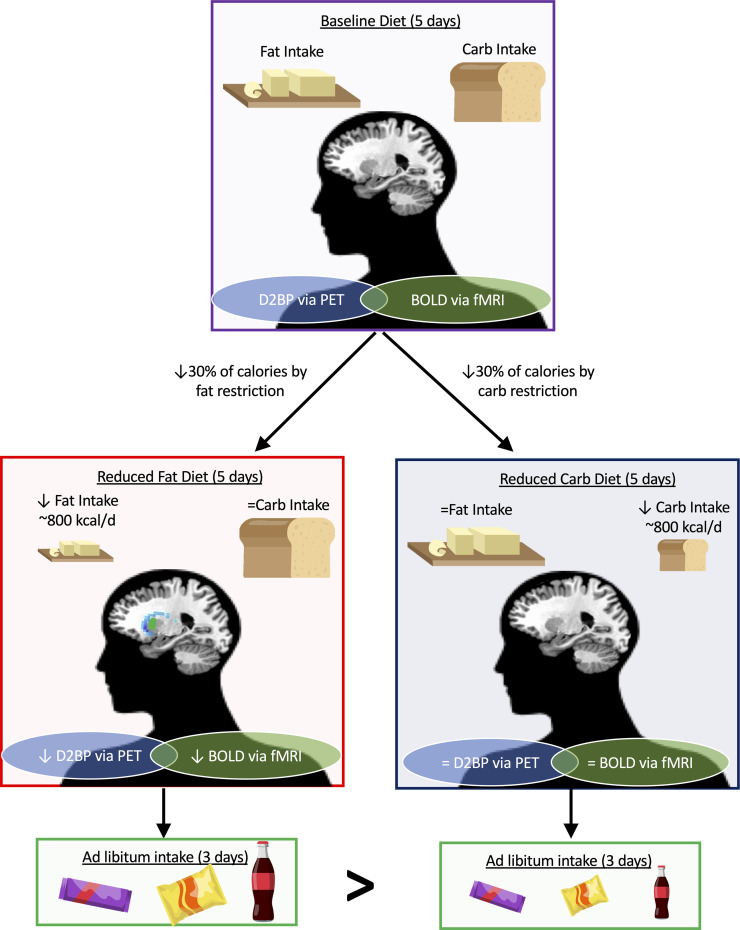

## Introduction

Among dietary approaches to treat obesity (1), popularity has waxed and waned between strategies that target dietary fat versus carbohydrates — macronutrients that elicit divergent peripheral metabolic and endocrine states (2). Dietary carbohydrate and fat ingestion also engages distinct gut-brain pathways affecting brain dopamine (3–5), which has been demonstrated in rodent models to be integral to eating behavior (6) and body weight regulation (7). While dopamine is fundamental to hedonic behaviors, the reinforcing properties of food are mediated only in part by the conscious sensory perception of pleasure per se. Rather, food reward is determined by signals originating predominantly from subconscious processes detecting nutritive cues to modulate dopamine signaling (8) in striatal regions involved in not only hedonic responses but also motivated behaviors, reinforcement learning, habit formation, and compulsion (6, 9). Thus, changes in brain dopamine may affect food choice and eating behavior.

People with obesity may have reduced dopamine synthetic capacity (10–12), and availability of striatal dopamine type 2/3 receptor binding potential (D2BP) may be correlated with adiposity (13–15). Brain dopamine has also been linked to human eating behavior (13, 16–18) and food reward processing (19) independently of body weight. Whether diets restricting carbohydrates versus fat differentially affect brain dopamine and eating behavior in humans is unknown. Here, we used positron emission tomography (PET) to measure D2BP and functional MRI (fMRI) to measure neural activity in response to visual food cues in 17 adults with obesity. Our prespecified objectives were to investigate whether 5 days of selective restriction of dietary fat or carbohydrates differentially affected D2BP and neural activity in response to visual food cues in brain-reward regions as compared with a eucaloric baseline diet.

## Results

**Figure 1:**
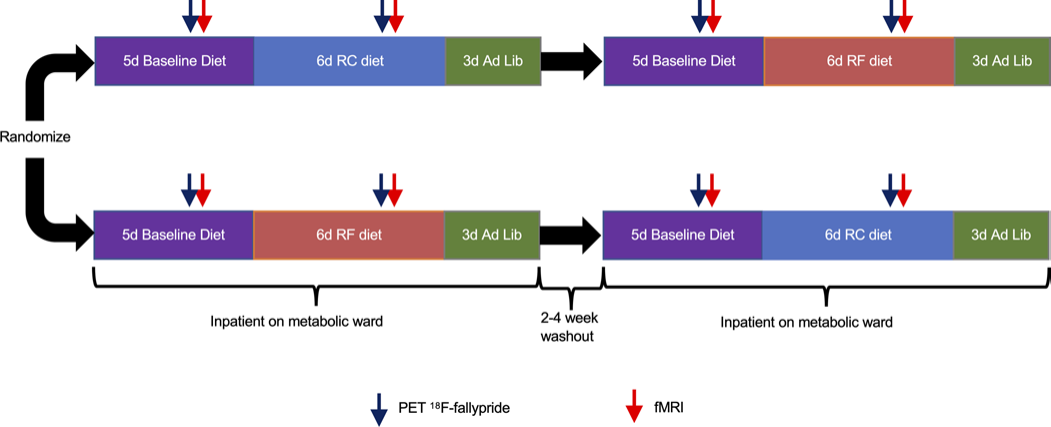
Study design. Seventeen men and women with obesity were admitted as inpatients to the Metabolic Clinical Research Unit at the NIH Clinical Center. They completed fMRI and PET scans on the third day of a 5-day inpatient eucaloric baseline diet, after which they were randomized to either a 30% reduced-calorie diet achieved by selective restriction of dietary fat (RF diet) or carbohydrate (RC diet). Neuroimaging was repeated on the fifth day of the reduced-energy diet, after which, on days 12–14 of the inpatient stay, participants consumed food ad libitum from vending machines. After a 2- to 4-week washout period, participants were readmitted as inpatients to complete the 5-day eucaloric baseline diet, and neuroimaging was repeated on the fifth day of the alternate 30% reduced calorie diet. During the final 3 inpatient days, participants again consumed food ad libitum from vending machines.

A subset of individuals for whom metabolic results were previously reported (2) included 8 male and 9 female weight-stable adults with obesity who were not currently on a restrictive diet (Table 1 and Supplemental Figure 1; supplemental material available online with this article; https://doi.org/10.1172/jci.insight.169759DS1) and had fMRI and PET neuroimaging at baseline and completed at least one of two 14-day visits to the Metabolic Research Unit at the NIH Clinical Center. As previously described (2), for 2 days prior to each inpatient admission, participants were asked to completely consume a provided standard eucaloric baseline diet (50% calories from carbohydrates, 35% calories from fat, 15% protein) that was continued for the first 5 days of admission. For the next 6 days, participants were randomized to consume a 30% calorie-restricted diet achieved either via selective reduction in fat (RF) or reduction in carbohydrates (RC), while keeping the other 2 macronutrients unchanged from the eucaloric baseline (Figure 1). For the final 3 days of each inpatient period, participants were given ad libitum access to vending machines stocked with a variety of supermarket foods. After a washout period of 2–4 weeks, participants were readmitted and consumed the eucaloric baseline diet for 5 days, followed by the alternate restricted diet for 6 days and ad libitum vending machine access for 3 days.

### Only the RF diet decreased activity in brain-reward regions in response to food cues.

Participants rated the pleasantness of a variety of food images during fMRI sessions 4.5 hours after lunch on the fifth day (third inpatient day) of the first eucaloric baseline diet period and on the fifth day of the RC and RF diets. Voxel-wise blood-oxygen-level-dependent (BOLD) responses to food images were compared with fixation within an a priori reward-region mask encompassing orbitofrontal cortex and striatal-pallidal neurocircuit as previously reported (20). On average, the restricted diets did not significantly impact explicit ratings of food pleasantness in the scanner (Supplemental Figure 2). 

**Figure 2:**
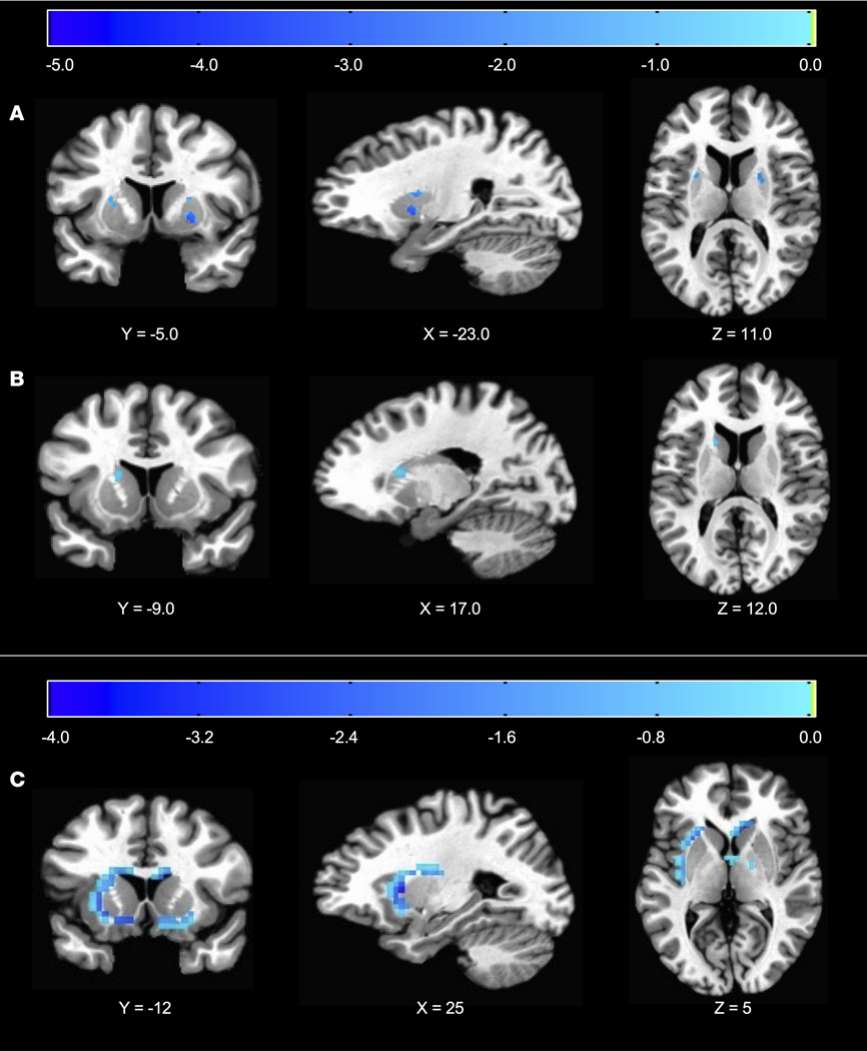
Selective reduction of dietary fat, but not carbohydrates, alters brain activity in reward regions. (**A** and **B**) Decreased striatal response to food cues using fMRI during the RF diet compared with both the baseline diet (**A**; *n* = 17) and RC diet (**B**; *n* = 15). (**C**) Reduced dopamine D2/3 receptor binding potential during the RF diet versus baseline using [18F]fallypride PET (*n* = 15). Corresponding cluster details are indicated in Table 2.

Compared with baseline, only the RF diet resulted in reduced activity in bilateral striatal clusters in caudate and putamen after correction for multiple comparisons as described in Methods (Figure 2A and Table 2). In contrast, the RC diet did not significantly change striatal responses to food images from baseline. Compared with the RC diet, the RF diet decreased activity in a dorsolateral region of the left caudate (Figure 2B and Table 2). Similar results were observed in the 15 participants with complete fMRI data for all diets (Supplemental Figure 3, A and B and Supplemental Table 1), as well as the subset of 13 participants who had complete fMRI and PET data (Supplemental Figure 3, C and D, and Supplemental Table 1). Unconstrained, whole-brain analyses confirmed that the reduction in striatal activity was limited to the RF diet compared with baseline and that the RF compared with the RC diet resulted in reduced activity distributed across prefrontal clusters (Supplemental Figure 4, A and B, and Supplemental Table 2). Whole-brain analysis of the RC diet compared with baseline revealed a significant increase in neural response to food cues in the posterior cingulate cortex (Supplemental Figure 4C and Supplemental Table 2).

### Only the RF diet led to decreased D2BP.

Participants completed PET imaging with the radiolabeled D2-like receptor subtype antagonist [^18^F]fallypride 2 hours after breakfast on the third inpatient day as well as on the fifth day of the RC and RF diets. [^18^F]fallypride time-activity curves using the cerebellum as a reference tissue were used with kinetic modeling to measure D2BP as previously described (13). A small volume correction (D2BP > 1.5) applied to whole brain analyses was used to isolate voxel-wise D2BP analyses to the striatum.

Compared with baseline, the RF diet significantly decreased D2BP in bilateral striatal clusters spanning the left putamen and right caudate/putamen (Figure 2C and Table 2). There was no significant effect of the RC diet on D2BP as compared with either baseline or the RF diet. Similar results were observed in the 15 participants with complete PET data during baseline, RF, and RC diets (Supplemental Table 1), as well as the subset of 13 participants with complete PET data who also had complete fMRI data (including 3 clusters surviving correction for multiple comparisons, α <0.05; Supplemental Figure 3E and Supplemental Table 1).

The cluster where D2BP was decreased during the RF versus the baseline diet was localized to the white/gray matter boundary of striatal nuclei. To rule out potential localization errors due to image misalignment, individual subject alignment data were visually checked independently by 2 members of the study team, and the mean group D2BP by diet condition was verified to map well with the template anatomical image in Talairach space (Supplemental Figure 5A). In our previously published PET processing and voxel-wise analysis pipeline (13), D2BP was calculated after PET data were linearly warped to Talairach space. To investigate whether cluster locations were related to the pipeline specifics, we also calculated D2BP in native space followed by nonlinear warping to Talairach space. Group level binding potential (BP) maps for each diet condition were calculated using this alternative pipeline and indicated that peak BP signal also appropriately mapped onto striatal gray matter (Supplemental Figure 5B), but the cluster location contrasting RF and baseline diets remained at the white/gray matter boundary (Supplemental Figure 6B).

### The RF diet resulted in greater ad libitum intake of foods high in both carbohydrate and fat.

We explored ad libitum food intake for 3 days after the RF and RC diets. Participants selected foods from computerized vending machines stocked with calories in excess of maintenance energy requirements. Average energy intake was (mean ± SEM) 25.9% ± 9.5% greater than the eucaloric baseline diet and was not significantly different following RF versus RC diets (Table 3). While overall macronutrient intake was similar after RC and RF diets, participants consumed a greater percentage of total calories from foods high in both carbohydrates and fat (HCHF) as well as high in both sugar and fat (HSHF) following the RF diet as compared with the RC diet (RF diet 28.8% ± 2.4% versus RC diet 23.1% ± 2.4%; *P* = 0.010) and consumed more calories from sugar-sweetened beverages (SSB) (RF diet 9.8% ± 1.1% versus RC diet 8.4% ± 1.1%; *P* = 0.032) such that the combination of HCHF, HSHF, and SSB as a fraction of total calories consumed was greater following the RF diet (RF diet 38.6% ± 3.0% versus RC diet 31.4% ± 3.0%; *P* < 0.001).

## Discussion

We previously showed that the RC diet led to widespread metabolic and endocrine changes compared with the eucaloric baseline diet, including increased fat oxidation as well as decreased energy expenditure and decreased daily insulin secretion, whereas the RF diet did not lead to substantial peripheral metabolic or endocrine changes (2). Therefore, we hypothesized that the RC diet would have a greater effect on brain-reward regions than the RF diet, especially given insulin’s previously reported effects on dopamine levels (21, 22). Surprisingly, it was the RF diet, and not the RC diet, that significantly decreased both D2BP and neural activity in response to visual food cues in brain-reward regions as compared with the baseline diet. The fMRI data show decreased activity in brain-reward regions in response to visual food cues during the RF diet as compared with the RC diet, but there were no significant differences in D2BP between RC and RF diets. Furthermore, ad libitum food intake after the RF diet was shifted toward high-fat, high-carbohydrate foods as compared with the RC diet. These results suggest that “a calorie is not a calorie,” when it comes to macronutrient effects on brain-reward regions in humans.

The most likely interpretation of our data is that the RF diet increased striatal tonic dopamine. This would explain the observed decrease in D2BP because increased endogenous dopamine would be expected to displace the [^18^F]fallypride tracer (23, 24). Furthermore, an increase in tonic dopamine would be expected to activate high-affinity D2-like receptors, thereby inhibiting neural activity (25) and explaining the observed decrease in brain activity to visual food cues with the RF diet. Indeed, pharmacological agonism of the D2-like receptor has been demonstrated to decrease the fMRI signal in both rats (26) and nonhuman primates (27).

D2 receptors are located both postsynaptically on nondopaminergic cells within the striatum and presynaptically on cell bodies, axons, and axon terminals of dopaminergic projection neurons (28). D2 receptors found presynaptically function as autoreceptors to modulate dopamine signaling (28) and are of relevance to control of human behavior (29). The RF diet decreased D2BP at the white/gray matter boundary of striatal nuclei, with a peak in apparent white matter, which might indicate differences in endogenous dopamine acting presynaptically on dopamine autoreceptors. Alternatively, localization to this region might have simply resulted from the limited resolution of PET to detect differences in D2BP at the edge of the striatum (30).

It is unlikely that the observed reduction in D2BP during the RF diet was due to decreased D2-like receptor density because neither dopamine depletion over 2–5 days (24, 31) nor dopamine stimulation over 5 weeks (32) appreciably impacts receptor density. Moreover, a reduction in D2-like receptor density would be expected to both minimize D2-like receptor inhibitory signaling and produce a net increase in the stimulatory effect of dopamine at neurons expressing D1/5 receptors, but this would be inconsistent with the observed decrease in fMRI response during the RF diet. Rather, the observed decrease in neural activity during the RF diet is consistent with increased tonic dopamine preferentially engaging inhibitory D2 receptor–expressing neurons with a high affinity for dopamine. Stimulation of activity in neurons expressing the lower-affinity D1/5 receptor requires phasic dopamine responses (33), which are expected to reduce — not increase — dopamine binding at the D2-like receptor (34). Phasic dopamine responses are not expected under our experimental conditions, given that the scans were conducted without providing rewards or reward-predicting stimuli. Indeed, visual food stimuli do not result in detectable changes in dopamine in humans (35, 36). Therefore, our multimodal neuroimaging results are most likely explained by increases in tonic striatal dopamine resulting from the RF diet.

An increase in tonic dopamine during the RF diet occurred in conjunction with an increased selection of high-fat, high-carbohydrate foods observed during the subsequent exploratory ad libitum period. Elevations in tonic dopamine alter the balance with phasic dopamine responses (33) and may increase incentive salience (37, 38), enhance the “wanting” of foods high in both carbohydrate and fat that are particularly rewarding (39, 40), promote selection of these foods previously experienced to deliver reward (41), and reduce the influence of any ensuing negative outcomes on changing behavior (42). It is intriguing to speculate about a role for tonic dopamine in influencing the food choices and making it difficult for people to adhere to low-fat diets, at least in the short term.

At first glance, our observation that a reduction in BOLD response to food cues during the RF diet occurred alongside a subsequent shift in ad libitum food selections toward high-fat, high-carbohydrate foods appears at odds with the literature on food cue reactivity suggesting a moderate positive association with subsequent weight gain and eating behavior (43). However, previous studies employed cross-sectional designs in participants consuming their habitual diets and did not experimentally manipulate the BOLD response to food cues. We speculate that decreased striatal BOLD response during the RF diet may be due to increases in tonic dopamine engaging inhibitory D2 receptor–expressing neurons, thereby biasing food choice toward rewarding foods.

How could reduction of dietary fat result in increased tonic dopamine in the brain? Dietary fat is detected and signaled to the brain throughout the alimentary canal from taste bud cells in the oral cavity to enteroendocrine and enterocyte cells in the gut (44). One of several mechanisms by which dietary fats modulate feeding includes intestinal production of oleoylethanolamide (OEA), a lipid messenger produced from dietary oleic acid that can signal to the brain via the vagus nerve (45–49). Despite OEA being produced from dietary fat, chronic consumption of high-fat diets in rodents decreases intestinal production of OEA and decreases brain dopamine (48). Because our study participants with established obesity reduced their fat intake by ~90 grams per day during the RF diet, their intestinal OEA production may have increased, thereby resulting in increased brain dopamine. In that case, the effect of increased intestinal OEA production might be expected to enhance satiety during the RF diet (45–49), while the increased tonic dopamine might have steered food choices away from such a diet toward more rewarding foods. In other words, adhering to a low-fat diet might be difficult, despite it potentially being more satiating and leading to decreased ad libitum energy intake in a setting where “off-diet” foods are unavailable (50).

Another potential mechanism for increased brain dopamine during the RF diet involves decreased postprandial plasma triglycerides that peak several hours after a meal in proportion to the amount of fat consumed (51). Triglycerides have been shown to suppress dopamine synthesis and excitability of D2-like receptor–expressing neurons (52) as well as to influence the preference for palatable food and reward-seeking in mice (53). Compared with the baseline and RC diets, the RF diet would be expected to result in reduced postprandial triglycerides and, therefore, to increase brain dopamine at the times of the neuroimaging scans conducted 2–3 hours postprandially.

Why did the RC diet have no significant effect on brain D2BP or neural activity in response to food cues, as compared with baseline? We found this result surprising particularly because the RC diet significantly decreased daily insulin secretion (2) and would be expected to decrease insulin in the brain (54), influencing multiple aspects of the dopamine system. For example, dopaminergic neurons express insulin receptors (55), and insulin decreases synaptic dopamine by increasing clearance from striatal synapses via enhanced dopamine transporter activity (56, 57). Consistent with a decrease in synaptic dopamine, intranasal insulin delivery was recently observed to increase D2BP in humans (21). Therefore, the lack of significant effect of the RC diet on brain dopamine remains a mystery. Whereas previous studies have demonstrated that calorie restriction potentiates dopaminergic signaling in both rodents and humans (58, 59), our results using 30% calorie-restricted RC and RF diets suggest that restriction of dietary fat may have a more potent effect on brain dopamine than isocaloric restriction of carbohydrates.

How might changes in brain dopamine in response to different diets relate more generally to body weight regulation? Recent mouse data suggest that the effects of brain dopamine may not be isolated to canonical hedonic pathways of food reward. For example, striatal dopamine can also influence downstream hypothalamic nuclei traditionally attributed to control homeostatic feeding and regulate body weight (3, 60), ultimately promoting intake of foods that cause obesity and devaluing foods that do not result in obesity (60). It is therefore intriguing to speculate that diet composition may contribute to altering the homeostatic body weight “set point” via changes in brain dopamine.

### Limitations.

While our interpretation of increased tonic dopamine is supported by relative pharmacokinetic properties of D1/5 and D2-like receptors, and literature on D2-like receptor PET occupancy and fMRI activity, we did not directly measure brain dopamine. Consumption of dietary fat elicits rapid dopaminergic response in reward regions (61, 62). While we observed an effect of reduced-fat diet at the D2 receptor via tonic levels of dopamine, it is possible that the relatively high proportion of dietary fat in the reduced carbohydrate diet may have influenced DA system via mechanisms dependent on D1/5 receptors not examined here. Future studies are needed to delineate the effect of exposure duration (single meal versus multiday), receptor subtype–specific effects (availability of D1/5 versus D2-like receptors after exposure) and subsequent effect on ad libitum eating behavior.

Ad libitum eating behavior subsequent to the 5-day period of dietary restriction supports our interpretation of increased incentive salience for rewarding foods after the RF diet. However, our study was not specifically powered to detect differences in this exploratory outcome, and analyses were not corrected for multiple comparisons.

Our interpretation of the effect of RC and RF diets on brain dopamine is limited to the early stages of initiating reduced-energy diets and does not address long-term changes or adaptations in neurochemistry or reward. Future studies should investigate changes in neurochemistry and reward activity in relation to diet composition over longer periods of weight loss. Furthermore, only adults with obesity were included in the present study. Adults without obesity appear to have a greater capacity to synthesize dopamine (11, 12). Whether eucaloric RC and RF diets would result in similar effects on D2BP and fMRI response to food images in adults without obesity or at risk of obesity is unclear.

Finally, the number of participants completing neuroimaging scans is relatively small. While we endeavored to collect data on 20 participants based on our prespecified power calculations, analyses ultimately were limited to *n* = 17 with available and usable neuroimaging data. Thus, while this study is limited in power, measurement reliability was greatly enhanced by the within-subject random-order crossover study design that tested the effects of specific dietary interventions relative to each participant’s own brain at baseline (63). Nevertheless, our findings warrant future replication in other demographic cohorts.

## Methods

### Experimental model and subject detail

Twenty-one adults provided informed consent to participate in a randomized crossover trial investigating the effects of selective isocaloric reduction of dietary fat versus carbohydrates on macronutrient metabolism, striatal D2BP, and neural activity in response to food stimuli in brain-reward regions (ClinicalTrials.gov, NCT00846040). Study details regarding the primary metabolic outcomes were reported elsewhere (2). In brief, right-handed nonsmokers between 18 and 45 years of age with a reported BMI greater than 30 kg/m^2^ (body weight < 350 pounds) were recruited from the Washington, D.C., metro area. All were free from diabetes, recent weight changes (more than 5 kg increase or decrease in the past 6 months), physical mobility impairments, past or present history of drug abuse, and neurological or psychiatric disorders (including eating disorders such as binge eating) as assessed by an abbreviated Structured Clinical Interview for the Diagnostic and Statistical Manual of Mental Disorders. Furthermore, participants were free from evidence of diseases or medications interfering with study outcomes, allergies to food or local anesthetics, evidence of regular excessive use of caffeinated drinks and alcohol, or strict dietary concerns (vegetarian or kosher diet). Premenopausal women were studied in the follicular phase for each inpatient visit and were excluded if they were pregnant or breastfeeding. Participants reported self-identified race and ethnicity at time of admission to the NIH Clinical Center.

### Study details

This study was conducted between February 13, 2009, and October 20, 2014. Volunteers were admitted to the NIH Clinical Center for a 14-day period to receive the eucaloric baseline diet for 5 days. After that, volunteers received either the RC or the RF diet for the next 6 days, followed by 3 days of ad libitum feeding from a computerized vending machine (Figure 1). Participants were readmitted after a 2- to 4-week washout period to repeat the 5-day eucaloric baseline diet, followed by 6 days of the alternate reduced calorie diet and 3 days of ad libitum feeding. Every day, participants completed 60 minutes of treadmill walking at a fixed self-selected pace and incline determined during screening to mimic free-living levels of physical activity.

The CONSORT diagram reiterates enrollment details provided in ref. 2 (Supplemental Figure 1). Two participants withdrew during the first baseline diet and did not complete any neuroimaging. Of the 19 participants who completed the initial baseline diet, 10 were randomized to next receive the RC diet and 9 were randomized to next receive the RF diet. The study team enrolled participants who were assigned to hypocaloric diet sequence via simple randomization conducted by the NIH Clinical Center Pharmacy, and diet interventions were implemented by the NIH Clinical Center Nutrition Department Staff. Among 10 participants receiving the RC diet on their first admission, 1 participant completed PET but not fMRI procedures during the RC diet and 2 withdrew before receiving the RF diet on the second planned admission. Among the 9 participants receiving the RF diet on their first admission, 2 completed fMRI but not PET on their first admission (participant-declined PET scans), and 1 participant did not have available fMRI data during their second admission on the RC diet. Full neuroimaging data (PET and fMRI) across all 3 diet conditions are available for *n* = 13 participants, and the results are provided in Supplemental Materials (Supplemental Figure 3, C–E, and Supplemental Table 1). Complete PET data are available in *n* = 15 participants. Complete fMRI data are available from *n* = 15 participants, and the results are provided in Supplemental Materials (Supplemental Figure 3, A and B, and Supplemental Table 1). 

### Anthropometrics

Height was measured in centimeters using a wall stadiometer (Seca 242), and weight was measured in kilograms using a digital scale (Scale-Tronix 5702). All measurements were obtained after an overnight fast, while participants were wearing only hospital scrubs.

### Diets

All subjects were confined to the metabolic ward throughout the study without access to outside food. Meals were consumed under observation, and any uneaten food was returned to the kitchen and reweighed. Subsequent meals were adjusted to account for uneaten food as needed. Diets were designed using ProNutra software (version 3.4, Viocare Inc.). Dietary interventions did not result in any adverse events, harm, or unintended effects in any condition.

#### Baseline eucaloric diet.

The daily caloric content during the initial out-patient segment and the weight-maintenance phase was based on the resting energy expenditure measured at screening with an activity factor of 1.5. Beginning 2 days before each admission, participants were provided with a weight-maintenance diet using a standard diet composition of 50% carbohydrate, 35% fat, and 15% protein, which continued for the next 5 days. All participants were provided with the standard diet during the first inpatient admission for at least 1 day prior to measuring baseline fMRI and D2BP. Energy and macronutrient intake during the baseline eucaloric diet are presented in Table 1.

#### Reduced energy diets.

During the restricted diet phase (inpatient days 6–11), 30% of baseline calories were removed by selective reduction of either carbohydrate (RC diet) or fat (RF diet) while keeping the other 2 macronutrients unchanged from eucaloric baseline diet. Energy and macronutrient intake during the reduced energy diets are presented in Table 1.

#### Ad libitum vending machine diet.

For the last 3 days of each inpatient stay, participants were given ad libitum access to a computerized vending machine (StarFood, Necta). The macronutrient self-selection paradigm procedure (MSSP) was used to select items for stocking the vending machine (64). This paradigm was selected for use in this study, since it was developed to show preference between foods of differing fat and carbohydrate content. It is composed of 6 categories of food, including HCHF, high complex carbohydrate/low fat (HCLF), low carbohydrate/high protein/high fat (HPHF), low carbohydrate/high protein/low fat (HPLF), HSHF, and high simple sugar/low fat (HSLF). A list of 76 foods that fit into these categories was provided to participants in a food questionnaire. This questionnaire contained Likert-type scales with questions in which the participant rated how much they liked each of the food items that could potentially be provided in the vending machine. The questionnaire also asked how often each of those food items were consumed normally by the participant (e.g., daily, weekly, or monthly). Of these foods, a total of 40 items that fit into the previously mentioned categories were chosen for inclusion in the vending machine, if preference was rated from 4–9 on the 10-point Likert scale.

Vending machines were stocked with traditional breakfast, lunch, dinner, and snack items. Beverages and condiments were also included in the vending machine, and consumption of these items was also recorded. SSB included fruit juices, lemonade, chocolate milk, and regular sodas. Each participant had access to 1 vending machine that only they could access. Once foods were selected, participants were instructed to eat in the dining area, and no food was allowed in the participant’s room. All uneaten food and wrappers were returned to the Metabolic Kitchen to be weighed. The vending machines were restocked daily at 8 a.m. with items that had been removed in the previous 24 hours. All foods were weighed to the nearest tenth of a gram on a digital scale (Mettler Toledo MS Series) prior to placing them in the computerized vending machine, and the remainder of any uneaten foods were weighed after consumption. Energy and macronutrient composition of the foods consumed from the vending machine were calculated using a computerized nutrition database (ProNutra, Viocare Inc.).

Vending machine foods were retrospectively categorized as either ultraprocessed or non-ultraprocessed based on NOVA categories (65) and were additionally categorized as hyperpalatable or nonhyperpalatable based on definitions presented in ref. 66.

Statistical analyses of caloric intake from Vending Machines were performed using IBM SPSS Statistics (28.0.1.1). Repeated-measure mixed model analyses were used to assess differences in intake of energy, macronutrients, and percent of calories from MSSP and SSB among 17 participants completing both 3-day ad libitum periods.

### MRI

On the afternoon following the morning PET scanning, high-resolution anatomical brain MRI was acquired with a HDx General Electric 3 Tesla scanner (echo time [TE] = 2.7ms, repetition time [TR] = 7.24 ms, flip angle 12°, voxel size 0.937 ***×*** 0.937 ***×*** 1.2mm) for each subject.

Under each diet condition, all subjects were scanned at 18:00, 4.5 hours after a standardized, diet-appropriate meal. Functional and structural imaging was performed on a 3T General Electric scanner and a GE 8-channel receive-only head coil. High-resolution anatomical images were collected prior to functional scanning runs (TE = 2.7 ms, TR = 7.24 ms, flip angle: 12°, voxel size: 0.937 × 0.937 × 1.2 mm). For the functional scans, 206 magnetic resonance (MR) volumes were acquired. Each echoplanar image (EPI) consisted of 44 2.8 mm slices (TE = 27 ms, TR = 2,500 ms, flip angle = 90°, voxel size = 3.4375 × 3.4375 × 2.8 mm). All structural and functional images were collected with a Sensitivity Encoding (SENSE) factor of 2 used to reduce image collection time (for structural images) or minimize image distortions (in functional images) while reducing gradient coil heating over the course of the scan session.

The fMRI task is described in detail elsewhere (20). In brief, 144 visual food cues ranging from highly processed, energy-dense foods to raw fruits and vegetables were displayed to participants using E-prime software (www.pstnet.com). Images projected to the scanner-room screen were viewed via a head coil–mounted mirror. Each image was presented for 5 seconds, during which time participants indicated their response to a question (“If given the opportunity right now, how pleasant would it be to eat this food?”) using an MR-compatible scroll wheel to select values along a number line positioned next to the image. A fixation cross was presented for varying durations between stimuli (mean interstimulus interval = 3.7 seconds; duration 2.5–7.5 seconds). The pleasantness rating scale ranged from 1 to 7, with 1 depicted as “neutral” and 7 as “extremely pleasant” and included an “unpleasant” option represented by the letter “X” located below the number line. For images that participants viewed as “unpleasant,” they were instructed to select the “X” if they believed the depicted food would be at all unpleasant to eat. Food images rated as “unpleasant” were excluded from the MRI and behavioral analyses.

Analyses of functional neuroimaging were performed in AFNI (AFNI_20.2.00 ‘Aulus Vitellius’). Each individual’s anatomical MRI was transformed into the Talairach space, and the transformation matrix was applied to the functional data during preprocessing. All functional volumes were aligned to a common base EPI represented by the third volume of the first functional run. The first 3 volumes of each EPI run were trimmed to allow the fMRI signal to reach steady state. A slice-time correction was applied to all functional volumes, which were also smoothed with a 6 mm full-width half-max Gaussian kernel. Additionally, the signal value for each EPI volume was normalized to the percent signal change from the voxel’s mean signal across the time course.

Individual subject data were checked for quality assurance, and outlying time points resulting from head motion were censored from the analyses. At the individual level, multiple regression was used to analyze the data, with regressors of noninterest included in the model to account for each run’s signal mean, linear, quadratic, and cubic signal trends, as well as 6 motion parameters (3 translations and 3 rotations) saved from the image registration step during preprocessing. The food pleasantness task regressor was constructed by convolving a box-car function with a width of 5 seconds beginning at the onset of the food image with a gamma-variate function to adjust the predictor variable for the delay and shape of the BOLD response. Given similarities in pleasantness ratings across diet conditions (Supplemental Figure 2), task pleasantness ratings were not included as parametric modulators of the hemodynamic response.

### PET

PET scanning was performed using a High Resolution Research Tomograph (HRRT; Siemens Healthcare) a dedicated brain PET scanner with a resolution of 2.5–3.0 mm and a 25 cm axial field of view. Transmission scanning was performed with a 137Cs rotating pin source to correct for attenuation. Two hours after a standard breakfast, a bolus of approximately 5 mCi of [18F]fallypride was infused i.v. using a Harvard pump. The specific activity was approximately 2,000 mCi/μmol at time of injection, and the radiochemical purity of the radiotracer was > 99%. PET emission data were collected starting at radiotracer injection over 3.5 hours, in 3 blocks separated by 2 10-minute breaks. Thirty-three volumes were acquired at times 0, 0.25, 0.5, 0.75, 1, 1.25, 1.5, 1.75, 2, 2.5, 3, 3.5, 4, 4.5, 5, 6, 7, 8, 9, 10, 12.5, 15, 20, 25, 30, 40, 50, 60, 90, 110, 130, 170, and 200 minutes. During each scan block, the room was quiet and dimly lit, and each subject was instructed to keep their head as still as possible, relax, and try to avoid falling asleep. The image reconstruction process corrected for head motion, which was tracked throughout each scan. Each scan consisted of 207 slices (slice separation = 1.22 mm). The fields of view were 31.2 cm and 25.2 cm for transverse and axial slices, respectively.

The PET images were aligned within each scan block with 6-parameter rigid registration using seventh-order polynomial interpolation, and each block was aligned to the volume taken at 20 minutes of the first block. The final alignments were visually checked, with translations varying by < 5 mm and the rotations by < 5 degrees.

### Statistics

Power calculations were based on cross-sectional data, not within-subject repeated measurements of D2BP change and fMRI response in response to dietary manipulation, which had not previously been measured. To detect a meaningful difference of effect size in D2BP (ratio of the expected mean and the standard deviation of the paired differences), with 20 subjects measured, we estimated that the study would achieve a power of 98% via the paired samples *t* test (α = 0.05, 2-tailed). For fMRI scanning, power calculations were based on the number of subjects needed to detect significant differences in fMRI activation between 2 randomized conditions in adults without obesity. Preliminary interindividual variability of fMRI activity during cognitive tasks in adults without obesity was estimated to be approximately 0.7%. Our aim was to detect fMRI signal changes larger than 0.5%. A minimum of 20 subjects with obesity were therefore required, assuming a Type I error (false positive rate) of 0.05 and 80% power.

fMRI images were included in AFNI’s 3dttest++ to identify clusters of significant effects of the diet condition (RF > Baseline for *n* = 17; RC > Baseline for *n* = 17; RF > RC for *n* = 15). Analyses using participants with complete neuroimaging data (fMRI and PET) across 3 diet conditions (*n* = 13) were analyzed via AFNI 3dANOVA (Supplemental Figure 3, C and D, and Supplemental Table 1). Since diet condition did not have a significant impact on food pleasantness ratings, analysis of brain activity to food pictures was not modulated by pleasantness ratings to maximize study power. Small volume corrections were implemented within the ROI defined by the orbitofrontal cortex, striatal-pallidal reward regions as previously described (20) with a voxel-wise *P* < 0.001 and a cluster size threshold (ke) > 5 voxels to achieve bisided correction for multiple comparisons at *P* < 0.05 via AFNI 3dClustSim.

Individual participants’ anatomical MRI images (see above) were coregistered to the aligned PET images by minimizing a mutual information cost function for each individual participant. For the analyses described in the main text, each individual’s anatomical MRI was linearly transformed into the Talairach space, and the transformation matrix was applied to the PET images, which were then smoothed with a 5 mm full-width, half-max Gaussian kernel. Data were exported to MATLAB where time-activity curves for [18F]fallypride concentration in each voxel were fit to a kinetic model (with the cerebellum used as the reference tissue) to determine D2BP (67). In an alternative pipeline presented in the Supplemental Materials, PET images were first smoothed and D2BP was calculated in native space followed by nonlinear warping to Talairach space.

Participants’ D2BP maps were included in AFNI’s 3dttest++ identify clusters with significant effects of diet (RF>Baseline for *n* = 15; RC>Baseline for *n* = 17; RF>RC for *n* = 15). Analyses using participants with complete neuroimaging data across 3 diet conditions (*n* = 13) were analyzed via AFNI 3dANOVA (Supplemental Figure 3E). Since high D2BP occurs mainly in striatum, small volume corrections were implemented within each hemisphere where D2BP >1.5. A bi-sided voxel-wise threshold of *P* < 0.1 was used, and cluster size threshold to achieve correction for multiple comparisons at *P* < 0.05. Using a full mixed effects model (AFNI 3dANOVA3), clusters survive correction for multiple comparisons using 3dClustSim at α of 0.05 a threshold of 33 voxels.

To test the robustness of our results with respect to alterations in processing and analysis pipeline, we also analyzed the data Individual participants’ anatomical MRI images were coregistered to the aligned PET images by minimizing a mutual information cost function for each individual participant. The aligned PET images were smoothed with a 5-mm full-width, half-max Gaussian kernel. Data were exported to MATLAB, where time-activity curves for [18F]fallypride concentration in each voxel were fit to a kinetic model with the cerebellum used as the reference tissue to determine D2BP (67). The values of D2BP were then imported back into individual native spaces to construct D2BP maps. Each individual’s anatomical MRI was mapped into the Talairach space with the AFNI program auto_warp.py and produced a nonlinear transformation function, which was then applied to transform each individual D2BP map into the Talairach space (Supplemental Figure 5B and Supplemental Figure 6B).

### Study approval

All study procedures were approved by the IRB of the National Institute of Diabetes & Digestive & Kidney Diseases. Written informed consent was received prior to participation, and compensation was provided.

## Author contributions

KDH, ABC, WKS, PH, and AM designed the research study. WKS, JAA, JEI, and ABC conducted experiments and acquired data. VLD, JG, ABC, IG, JAA, and WKS analyzed data and performed statistical analysis. VLD and KDH drafted the manuscript. All authors contributed intellectually and approved the manuscript.

## Supplementary Material

Supplemental data

ICMJE disclosure forms

Supporting data values

## Figures and Tables

**Table 1:**
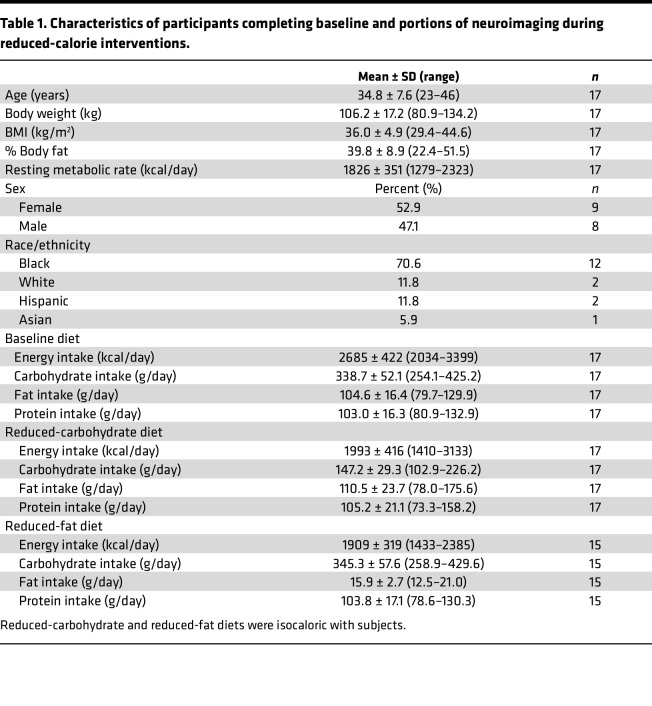
Characteristics of participants completing baseline and portions of neuroimaging during reduced-calorie interventions.

**Table 2:**
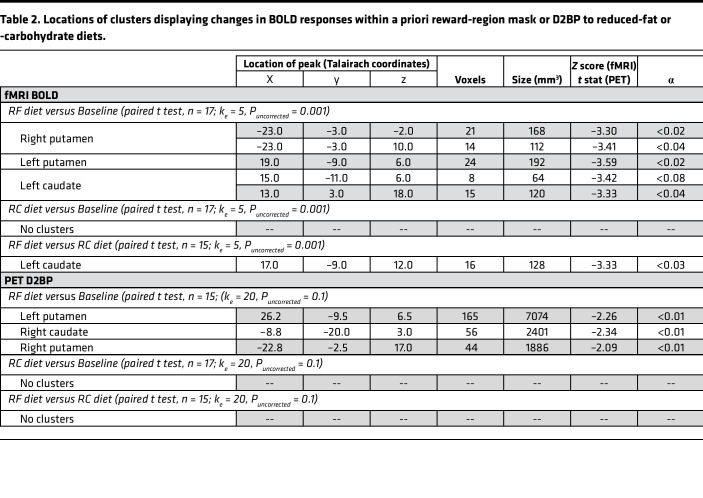
Locations of clusters displaying changes in BOLD responses within a priori reward-region mask or D2BP to reduced-fat or -carbohydrate diets.

**Table 3:**
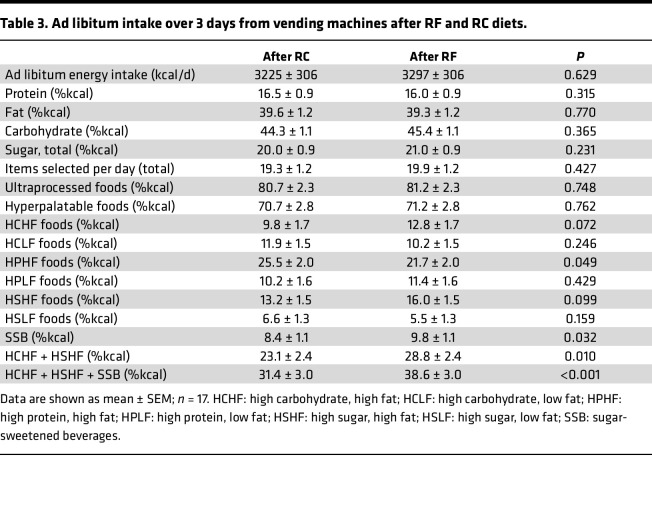
Ad libitum intake over 3 days from vending machines after RF and RC diets.

## Version 1 06/22/2023

Electronic publication
